# Using Electronically Delivered Therapy and Brain Imaging to Understand Obsessive-Compulsive Disorder Pathophysiology: Protocol for a Pilot Study

**DOI:** 10.2196/30726

**Published:** 2021-09-14

**Authors:** Callum Stephenson, Niloufar Malakouti, Joseph Y Nashed, Tim Salomons, Douglas J Cook, Roumen Milev, Nazanin Alavi

**Affiliations:** 1 Centre for Neuroscience Studies Faculty of Health Sciences Queen's University Kingston, ON Canada; 2 Department of Psychiatry Faculty of Health Sciences Queen's University Kingston, ON Canada; 3 School of Rehabilitation Therapy Faculty of Health Sciences Queen's University Kingston, ON Canada; 4 Department of Medicine School of Medicine Queen's University Kingston, ON Canada; 5 Department of Psychology Faculty of Arts and Science Queen's University Kingston, ON Canada; 6 Neurosurgery Division, Department of Surgery School of Medicine Queen's University Kingston, ON Canada

**Keywords:** mental health, obsessive-compulsive disorder, cognitive behavioral therapy, exposure ritual prevention, electronic, functional magnetic resonance imaging, eHealth, brain imaging

## Abstract

**Background:**

Obsessive-compulsive disorder (OCD) is a debilitating and prevalent anxiety disorder. Although the basal ganglia and frontal cortex are the brain regions that are most commonly hypothesized to be involved in OCD, the exact pathophysiology is unknown. By observing the effects of proven treatments on brain activation levels, the cause of OCD can be better understood. Currently, the gold standard treatment for OCD is cognitive behavioral therapy (CBT) with exposure and response prevention. However, this is often temporally and geographically inaccessible, time consuming, and costly. Fortunately, CBT can be effectively delivered using the internet (electronically delivered CBT [e-CBT]) because of its structured nature, thus addressing these barriers.

**Objective:**

The aims of this study are to implement an e-CBT program for OCD and to observe its effects on brain activation levels using functional magnetic resonance imaging (MRI). It is hypothesized that brain activation levels in the basal ganglia and frontal cortex will decrease after treatment.

**Methods:**

Individuals with OCD will be offered a 16-week e-CBT program with exposure and response prevention mirroring in-person CBT content and administered through a secure web-based platform. The efficacy of the treatment will be evaluated using clinically validated symptomology questionnaires at baseline, at week 8, and after treatment (week 16). Using functional MRI at baseline and after treatment, brain activation levels will be assessed in the resting state and while exposed to anxiety-inducing images (eg, dirty dishes if cleanliness is an obsession). The effects of treatment on brain activation levels and the correlation between symptom changes and activation levels will be analyzed.

**Results:**

The study received initial ethics approval in December 2020, and participant recruitment began in January 2021. Participant recruitment has been conducted through social media advertisements, physical advertisements, and physician referrals. To date, 5 participants have been recruited. Data collection is expected to conclude by January 2022, and data analysis is expected to be completed by February 2022.

**Conclusions:**

The findings from this study can further our understanding of the causation of OCD and help develop more effective treatments for this disorder.

**Trial Registration:**

ClinicalTrials.gov NCT04630197; https://clinicaltrials.gov/ct2/show/NCT04630197.

**International Registered Report Identifier (IRRID):**

PRR1-10.2196/30726

## Introduction

### Background

Obsessive-compulsive disorder (OCD) is a debilitating anxiety disorder that can manifest in a multitude of ways in individuals. The 1-year prevalence of OCD in Canada is estimated to be 1%, with approximately 3% of the population developing OCD at some point in their lifetime [1,2]. Although OCD is a relatively widespread disorder, there have been no conclusive results to date regarding its etiology [3,4]. By understanding the pathophysiology of OCD better, more targeted treatments can be developed, leading to higher effectiveness in symptom reduction.

OCD is categorized as having obsessions and compulsions that an individual is unable to control. These obsessions, uncontrollable thoughts, impulses, and ideas are found to be disturbing and anxiety-inducing by the individual. These obsessive thoughts and/or feelings typically manifest in the impaired control of mental activities, thoughts related to uncertainty, sex, violence, or contamination [5]. The differentiation between obsessive thoughts and common worries is that obsessive thoughts tend to be more unrealistic and involve more imagery than common worries [6]. Moreover, individuals are often embarrassed by their obsessive and intrusive thoughts and can be reluctant to share these thoughts with their health care provider [7]. Individuals with OCD often combat their obsessive thoughts with compulsive, uncontrollable behaviors. These repetitive behaviors or cognitive acts are intended to reduce the individual’s anxiety. Common compulsions include hand washing, and checking and rigid maintenance of order and organization. Common cognitive acts include counting numbers, praying, and repeating words or phrases over and over. Much as with their obsessive thoughts, individuals with OCD are commonly embarrassed by their compulsions and recognize that they are irrational but are still unable to refrain from them. Although obsessions can occur exclusively without compulsions, it is more likely that they co-occur [8]. Individuals with OCD may also use neutralizations, which are repeated thought processes that help prevent, cancel, or reverse the feared consequences and distresses caused by their obsession [9,10]. Individuals with OCD often present with a concern that their thoughts will lead to negative consequences for themselves and/or others. An additional common trait in individuals with OCD is thought-action fusion, the belief that thinking of an event increases the probability of its occurrence and that a thought is a moral equivalent of the physical act [11,12].

Cognitive behavioral theories explain the etiology of OCD, with obsessive behaviors serving as a distraction from unpleasant thoughts [10]. It is theorized that abnormal obsessions are caused by problematic reactions to feelings of elevated responsibility, leaving the individual feeling a need to always be competent to avoid criticism. The compulsive behaviors then serve as an avoidance tactic [9,13]. These compulsions reinforce maladaptive behaviors and eventually develop to outlast the cause of the anxiety-inducing thought [14]. By using these theories and observing the effect of treatment on cognitive anxiety processes at a neural level, we can understand the etiology of OCD better.

Cognitive behavioral therapy (CBT) with exposure and response prevention (ERP) is regarded as the first-line treatment for OCD [15-20]. CBT targets thoughts and cognition, the central components of OCD [20,21]. The structure of CBT for OCD is similar to that of CBT for depression or anxiety, with the addition of ERP being the biggest difference [22-24]. Although CBT is a frontline treatment, it is costly, time consuming, and often inaccessible. Therefore, more accessible and efficient interventions must be developed without sacrificing the quality of care. Fortunately, web-based mental health interventions have become increasingly popular, and the electronically delivered CBT (e-CBT) is a feasible option [25]. Research suggests that e-CBT is an effective and feasible intervention for OCD, with results comparable with those of in-person treatment [25-30]. However, e-CBT is often implemented in nonscalable and nonsecure formats. Moreover, the efficacy of CBT, along with new and innovative treatments, can be drastically improved if we understand better how treatment affects neural anxiety processing and cognitive functioning [16-21].

The most implicated regions of the brain associated with OCD are the basal ganglia and frontal cortex. These regions are involved in motor control and cognitive functioning tasks, including abstract reasoning, planning, decision-making, and inhibition [31-33]. More specifically, the basal ganglia play a large part in selecting movements that have positive effects [34]. In the case of OCD, it is hypothesized that this could play a part in the compulsive behaviors of OCD (eg, hand washing if cleanliness is an obsession). The frontal cortex is involved in reward processing, impulse control, and emotional control, among others [35]. If we learn that treatment can alter a patient's anxiety processing at a neural level, more specific treatments targeting these brain regions can be developed. Research suggests that in patients with OCD, the basal ganglia and frontal cortex are hyperactive at rest, becoming further activated while in anxiety-inducing situations [13,15,33,36-43]. These studies also suggest that successful treatment may result in a partial reversal of this hyperactivation [13,15,33,36-43]. However, more work is needed to make definitive conclusions on whether activation levels decrease following successful treatment.

By measuring brain activation levels while exposed to neutral and anxiety-inducing stimuli pre- and posttreatment, the suspected regions involved in OCD pathology (basal ganglia and frontal cortex) can be observed. These findings can help explain whether effective treatment results in changes in activation levels, whether symptom improvement is correlated to changes in activation levels, and if there are any treatment response identifiers. If anxiety processing in patients with OCD can be understood better, more targeted treatments altering these specific parts of cognition can be developed. It is currently unknown whether e-CBT can produce these effects.

### Objectives

This study will use a 16-week e-CBT with an ERP program to evaluate the effects of treatment on activation levels in the basal ganglia and frontal cortex. Previous findings suggest that CBT may partially reduce the hyperactivation commonly observed in OCD. It is hypothesized that successful treatment will decrease activation levels in the basal ganglia and frontal cortex when comparing pre- and posttreatment levels in patients with OCD with controls. This study will aim to address the following questions:

1. Does successful treatment result in changes in brain activation levels in regions involved in neural anxiety processing at the core of OCD pathology?
2. Is there a correlation between changes in symptom severity and changes in brain activation levels after treatment in patients with OCD?
3. What is the efficacy of this e-CBT program compared with control in improving symptoms, quality of life, and levels of functioning in patients with OCD?
4. What is the feasibility and user experience of this e-CBT program from a patient perspective?

## Methods

### Design

A nonrandomized pilot study design will be used with all participants receiving 16 weekly sessions of e-CBT. Functional magnetic resonance imaging (fMRI) will be conducted at baseline and posttreatment to evaluate activation level changes in the basal ganglia and frontal cortex. Clinically validated symptomology questionnaires will be used to evaluate treatment efficacy. In addition, qualitative interviews will be conducted to gather personal demographic information as well as information regarding participant experience while using the web-based psychotherapy clinic. The pilot study has been registered on the ClinicalTrials.gov Protocol Registration and Results System (NCT04630197). In addition, ethics approval has been obtained from the Queen’s University Health Sciences and Affiliated Teaching Hospitals Research Ethics Board (HSREB; file number 6031276).

### Participants

Participants (n=10) will be recruited from family medicine and psychiatric clinics at Queen’s University and Kingston Health Sciences Centre sites (Hotel Dieu Hospital and Kingston General Hospital) in Kingston, Ontario, Canada. In addition, local and social media advertisements will be used. Participants will be enrolled in the study based on referrals from outpatient clinics and family doctors as well as self-referrals. Those invited and interested in participating will have the study protocol explained and an evaluation done by a psychiatrist on the research team through a secured video appointment. Participants will be evaluated for a diagnosis of OCD based on the *Diagnostic and Statistical Manual of Mental Disorders, 5th Edition* [44]. After a diagnosis of OCD is confirmed and the participant is given written and verbal instructions on how to participate in the study, informed consent will be obtained.

The inclusion criteria include the following: between the ages of 18 and 65 years at the start of the study, a diagnosis of OCD according to the *Diagnostic and Statistical Manual of Mental Disorders, 5th Edition* criteria, competence to consent to participate, ability to speak and read English, and consistent and reliable access to the internet. Exclusion criteria include the following: having any metal implants or additional factors deemed not safe for an MRI scan, active psychosis, acute mania, severe alcohol or substance use disorder, and/or active suicidal or homicidal ideation. In addition, if a participant is currently receiving another form of psychotherapy, they will be excluded from the study.

### Therapy

Weekly sessions of e-CBT will be conducted through the Online Psychotherapy Tool (OPTT; OPTT Inc), a secure, web- and cloud-based mental health care delivery platform. These web-based sessions will consist of approximately 30 slides and interactive therapist videos, with 16 modules in total (1 module per week). The content and format of these web-based sessions will mirror in-person CBT for OCD. The connection between thoughts, behaviors, emotions, physical reactions, and the environment will be a focus. Moreover, mindfulness, body scanning, self-care, goal setting, thinking errors, the 5-part model, and thought records will be employed as techniques for participants. ERP will be incorporated into the e-CBT program as this is the first-line route of treatment. Slides will highlight different topics each week and include general information, an overview of skills, and homework on that topic. The homework included in each session will be submitted through OPTT and reviewed by therapists, with personalized feedback provided within 3 days of submission. Weekly homework submission for feedback will be mandatory before being eligible for the next session. After each completion of the e-CBT program, participants will be interviewed to investigate their experience using OPTT and their perceptions of how the treatment went. OPTT can be accessed from a variety of devices (ie, desktop computers, laptops, cell phones, and tablets) and internet browsers.

### Imaging

All imaging will occur at the Queen’s University MRI Facility in Kingston, Ontario, Canada, using a Siemens 3.0 Tesla whole-body MRI scanner with a standard coil. Scans will occur at baseline (pretreatment) and after week 16 (posttreatment). During scanning, participants will lie on the scanning table on their backs, with their heads resting on a foam pad to reduce movement. Scanning appointments will take approximately 1 hour per session.

Anatomical reference images will be captured initially. After this, fMRI scans will occur while participants are shown neutral images and anxiety-inducing images (eg, dirty dishes if cleanliness is an anxiety-inducing concept for a specific participant). The frontal cortex and basal ganglia will be the focus of the imaging procedures as their activation level changes during neural anxiety processing are of interest. These images will be standardized pictures from the International Affective Picture System [45]. This large database allows for the selection of images that relate to a variety of obsessions representative of each participant (ie, contamination, sexual thought intrusion, and fear of harm). Each set of pictures will be individually tailored to each participant. These images will be selected ahead of time by a psychiatrist on the research team and will be related to the participant’s anxieties. Participants will be shown a total of 40 images (20 neutral and 20 anxiety inducing; R=0.5) during the fMRI sessions. There will be four fMRI runs that occur in the following sequence:

1. 5 neutral images (30 seconds per image, 5 seconds break between), 1-minute break, 5 anxiety-inducing images (30 seconds per image, 5 seconds break between), 1-minute break.
2. 5 new anxiety-inducing images (30 seconds per image, 5 seconds break between), 1-minute break, 5 new neutral images (30 seconds per image, 5 seconds break between), 1-minute break.
3. 5 new anxiety-inducing images (30 seconds per image, 5 seconds break between), 1-minute break, 5 new neutral images (30 seconds per image, 5 seconds break between), 1-minute break.
4. 5 neutral images (30 seconds per image, 5 seconds break between), 1-minute break, 5 anxiety-inducing images (30 seconds per image, 5 seconds break between).

The images will be shown in sets (groups of 5 images) as opposed to intermingled, in the hope of producing a more sustained emotional state and allowing for more distinct readings. The ordering of the image sets repeats halfway through (back to back of the anxiety-inducing images in the example above) to control for participants becoming accustomed to image ordering. This ordering will be changed for every participant (ie, the next participant would receive back-to-back sets of the neutral imaging in runs 2 and 3) to counterbalance the imaging sets. Participants will be prompted to imagine themselves in the situations described in the images. The images will appear on a screen that will be reflected into the scanner for participants to view. A 0.5% blood oxygen level–dependent (BOLD) signal difference between conditions (*P*<10^-6^) will be considered a detectable change (eff=0.005).

Anatomical reference images will be captured with the phase-encoding direction collected sagittally from anterior to posterior. These images will be captured with T1-weighted high-resolution magnetization-prepared rapid acquisition gradient-echo (MPRAGE) images with 0.8×0.8×0.8 mm^3^ isotropic voxels. These images will use a 256 mm field of view (FOV), 2500 ms repetition time (TR), 2.22 ms echo time (TE), 8-degree flip angle, and a 320×320 mm matrix resolution. Following this, T2*-weighted gradient-echo echo-planar imaging (GE-EPI) with 3.0 mm 3.0×3.0×3.0 mm^3^ isotropic voxels will be used for the stimuli-exposed image acquisitions in an anterior-to-posterior direction. These images will use a 192 mm FOV, 2500 ms TR, 28.4 ms TE, 90-degree flip angle, and a 64×64 mm matrix resolution. A multiband acceleration factor of 2 will be used, with 170 volumes being captured. Following the GE-EPI imaging, two short spin-echo field map scans will be captured from anterior to posterior and then posterior to anterior. These images will use a 192 mm FOV, 8000 ms TR, 66.0 ms TE, 90-degree flip angle, 180-degree refocus flip angle, and a 64×64 mm matrix resolution. All images will use a bandwidth of 1500 Hz.

The GE-EPI fMRI data will be mapped to a nondistorted set of gradient-echo images from the same participant to undistort the images. Next, the nondistorted gradient-echo images will be mapped onto the T1-weighted MPRAGE image. Finally, the T1-weighted MPRAGE will be mapped to the Montreal Neurological Institute standardized brain template. In doing this, the GE-EPI fMRI data will be mapped to the Montreal Neurological Institute template with maximum accuracy.

### Training

All therapists will be research assistants trained in psychotherapy delivery and supervised by a psychiatrist on the research team who has extensive experience in electronically delivered psychotherapy. All therapists are taught the standard care pathway, the aim, and the content of each therapeutic session. Moreover, they will be provided with sample homework from a previous patient and will be asked to provide feedback as practice. Feedback templates will vary between sessions, and therapists will personalize each template for each patients’ homework. Before feedback is submitted to the participant, it will be read, edited, and approved by a psychiatrist on the research team. Training will occur through webinars and exercises with feedback.

### Outcomes

The primary outcome measure will be changes in activation levels of the basal ganglia and frontal cortex. This will be collected through detectable changes in BOLD values from the fMRI scans at baseline and after treatment (week 16). The secondary outcomes will be changes in symptom severity, quality of life, and functioning. Changes in symptom severity will be evaluated using clinical symptomatology questionnaires (Yale-Brown Obsessive-Compulsive Scale and Obsessive-Compulsive Inventory, Revised) [46,47]. Changes in quality of life will be measured using the Quality of Life and Enjoyment Questionnaire [48]. Changes in levels of functioning will be measured using the Sheehan Disability Scale [49]. All questionnaires will be collected directly through OPTT at baseline, after session 8, and after treatment (week 16).

### Compliance

As with all mental health disorders, treatment compliance is always an area of focus when designing interventions. Participants will have the importance of treatment compliance explained to them during the informed consent process, and participants will need to submit their homework assignments through OPTT before gaining access to their next treatment session. From a previous meta-analysis conducted, the estimated completion rate from in-person psychotherapy is approximately 75% [50]. Additional meta-analyses found treatment adherence for web-based psychotherapy to be between 61% and 66%, with no significant difference with in-person psychotherapy [51-53]. A study investigating the efficacy of a 10-session e-CBT program for OCD had a mean completion of 7.28 sessions [54]. Previous research using OPTT indicated participants completed >8 sessions on average, with over half the participants completing all sessions. In a previous project using e-CBT for patients with generalized anxiety disorder, 90% of participants completed 10-12 weeks of the 12-week program, with over 75% of participants being retained for a 12-month follow-up [55].

### Analysis

For the fMRI data (primary outcome), a 0.5% (eff=0.005) change in BOLD hemodynamic response function will be considered a detectable signal variation between conditions (*P*>10^-6^). An estimated paradigm of expected BOLD response will be created, and the correlation between the real and expected signals will be calculated to detect noise using a general linear model:

S = βX + e **(1)**

Where, S is the time-series data, β is the value for each pattern, X is the set of time-series patterns, and e is the residual.

The general linear model will provide a β value for each term in the basis set and a T value for each β. The BOLD contrast between conditions and scanning periods (ie, baseline and posttreatment) will be evaluated using one- and two-sample *t* tests, assuming a normal distribution. Realignment parameter regressors for the testing conditions will be implemented [56,57]. The effects at each condition will enter a group analysis using a random-effects model [58]. A group-level comparison will be used with small volume corrections performed for multiple comparisons using the Gaussian random field theory. Missing data points can be accounted for in the analysis with usable questionnaires and fMRI data using the linear model.

For questionnaire scores (secondary outcomes), a linear regression analysis will be used to identify variables associated with the outcome measures while controlling for demographic variables (ie, age, sex, and gender). Repeated measures of analysis of variance will be conducted to determine changes between periods (ie, baseline, week 8, and posttreatment) questionnaire scores. Greenhouse-Geisser adjustment for F-statistics and Bonferroni corrections for multiple comparisons will be used. Using the Pearson correlation coefficient, the correlation between the questionnaire score and the BOLD response will be evaluated. Data outliers will be defined as SD 3.29 away from the mean on scores.

Skew and kurtosis will be analyzed assuming a normal distribution in the questionnaire and fMRI data at all collection time points. Age, gender, and sex variables will be considered in knowledge creation and translation.

### Ethics and Privacy

The pilot study has received approval from the Queen’s University HSREB. Only the care providers involved in the care of the participants will have access to their information. Participants will only be identifiable by an identification number on the OPTT platform, and hard copies of consent forms with participant identity will be stored securely on site and will be destroyed 5 years after study completion. Only anonymized data will be provided to the analysis team members. OPTT is compliant with the Health Insurance Portability and Accountability Act, Personal Information Protection and Electronic Documents Act, and Service Organization Control–2. In addition, all servers and databases will be hosted in the Amazon Web Service Canada cloud infrastructure, which is managed by Medstack to assure all provincial and federal privacy and security regulations are met. OPTT will only collect anonymized metadata to improve its service quality and provide advanced analytics to the research team. OPTT will encrypt all data, and no employee will have direct access to patient data. All encrypted backups will be kept in the S3 storage dedicated to Queen’s University.

## Results

The pilot study is currently being conducted at Queen’s University by the members of the research team. This pilot study is a nonrandomized, open-label study, with a plan for an expanded randomized controlled trial in the future. The pilot study received ethics approval from the Queen’s University HSREB in December 2020, and the recruitment of participants began in January 2021. To date, 5 participants have been seen for initial assessments and enrolled in the study. Data collection is anticipated to begin in June 2021, with collection and analyses concluding by January and February 2022, respectively. All reporting of this study has been and will be in accordance with the GUIDance for the rEporting of intervention Development (GUIDED) and Template for Intervention Description and Replication (TIDieR) reporting checklists and guides (Multimedia Appendices 1 and 2).

## Discussion

This trial can contribute to creating more effective treatments for OCD in the future. However, to do this, we must understand the pathophysiology and etiology of this debilitating and prevalent condition. Currently, there is no conclusive information on the pathophysiology and etiology of OCD. Although CBT with ERP is currently the frontline psychotherapy for OCD, the efficacy of this treatment, along with those of new and innovative ones, can be improved drastically if we understand better how treatment affects neural anxiety processing and cognitive functioning. By measuring brain activation levels during exposure to neutral and anxiety-inducing stimuli pre- and posttreatment, we can observe how the suspected regions involved in OCD pathology (basal ganglia and frontal cortex) change following treatment. This can enlighten us on various aspects of OCD, including whether effective treatment results in changes to activation levels, whether symptom improvement is correlated to changes in activation levels, and if there are any identifiers for brain activation levels for responders and nonresponders to treatment. If how patients with OCD process anxiety-inducing stimuli (ie, dirty dishes if cleanliness is a patient's obsession) can be understood better, more targeted treatments can be given to specifically alter these parts of a person’s cognition.

## Appendix

Multimedia Appendix 1 GUIDED (Guidance for the Reporting of Intervention Development) report checklist.

Multimedia Appendix 2 TIDieR (Template for Intervention Description and Replication) report checklist.

## Abbreviations

BOLD: blood oxygen level–dependent
CBT: cognitive behavioral therapy
e-CBT: electronically delivered cognitive behavioral therapy
ERP: exposure and response prevention
fMRI: functional magnetic resonance imaging
FOV: field of view
GE-EPI: gradient-echo echo-planar imaging
GUIDED: GUIDance for the rEporting of intervention Development
HSREB: Health Sciences and Affiliated Teaching Hospitals Research Ethics Board
MPRAGE: magnetization-prepared rapid acquisition gradient-echo
OCD: obsessive-compulsive disorder
OPTT: Online Psychotherapy Tool
TE: echo time
TIDieR: Template for Intervention Description and Replication
TR: repetition time
